# Supplementary material for: Molecular “Yin-Yang” Machinery of Synthesis of the Second and Third Fullerene C60 Derivatives
Source: Micromachines (Basel). 2025 Jun 30;16(7):770. doi: 10.3390/mi16070770 (PMC12299826; doi:10.3390/mi16070770)
Supplement: Supplementary file 1 [file micromachines-16-00770-s001.zip › micromachines-3699158-supplementary.pdf]

micromachines

MDPI

Supplementary Materials

Molecular "Yin-Yang" Machinery of Synthesis of the Second and Third Fullerene C_{60} Derivatives

Djuro Lj. Koruga [1,*], Lidija R. Matija [1], Ivana M. Stanković [1], Vladimir B. Pavlović [2] and Aleksandra P. Dinić [1]

[1] NanoLab, Biomedical Engineering, Faculty of Mechanical Engineering, University of Belgrade, 11120 Belgrade, Serbia; lmatija@mas.bg.ac.rs (L.R.M.); imileusnic@mas.bg.ac.rs (I.M.S.); adinic@mas.bg.ac.rs (A.P.D.)
[2] TEM Laboratory, Faculty of Agriculture, University of Belgrade, 11000 Belgrade, Serbia; vlaver@agrif.bg.ac.rs
* Correspondence: dkoruga@mas.bg.ac.rs or djuro.koruga@gmail.com; Tel.: +381-63287353

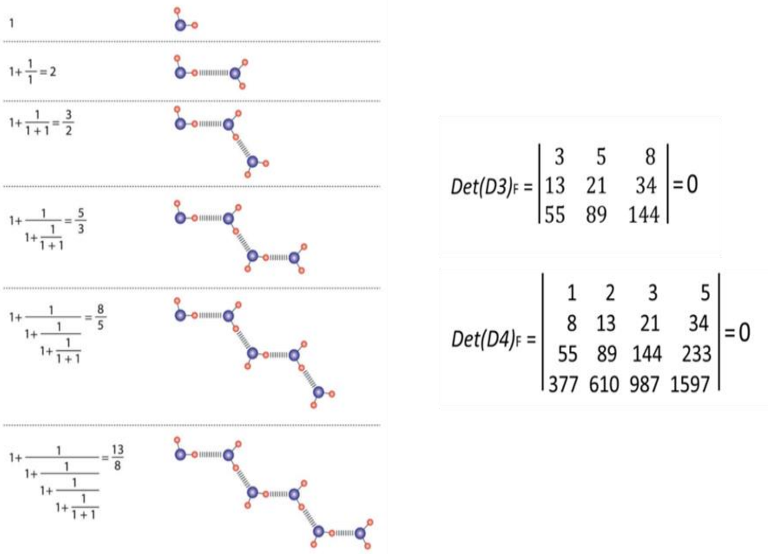

Figure S1: Order of water hydrogen bonds. The Fibonacci fractions values for chains of water molecules are 1,2,3/2, 5/3,8/5, and 13/8, respectively. These values are a set of Fibonacci sequences of Φ. The question is, what is the chain length of stable water molecules? The system of Fibonacci determinants provides the answer. The number of molecules where the determinant of non-covalent hydrogen bonds equals zero gives a stable chain. A chain of 2,3,4,5,6.7… water molecules would not be stable; it would be created and destroyed within a few ps. However, for a chain in which water molecules are part of the Fibonacci determinant, such a system (D3, nine chains, with 144 maximal number of water molecules of chain) is equal to zero, and the chains are stable and incoherent. The same case is true for the Fibonacci D4 systems with 16 chain types (1597 maximal water molecules in the chain). [Matija LR, Stankovic IM, Puric M, et al. The Second Derivative of Fullerene C_{60} (SD-C_{60}) and Biomolecular Machinery of Hydrogen Bonds: Water-Based Nano-medicine. *Micromachines* **2023**, *14*, 2152. https://doi.org/10.3390/mi14122152].

Table S1: Icosahedral symmetry group that determines the energy states (T_{1g}, T_{2g}, T_{1u}, and T_{2u}) of structures and processes. Fibonacci numbers Φ, -ϕ, -Φ, ϕ are subsets of the icosahedral symmetry group (Φ= ½(1+ √ 5) and -ϕ = ½(1- √ 5)) (Adapt from Kettle, S.F.A., Symmetry and structure, John Willey and Sons, Chichester, 1995).

I/I_h	E	$12C_5$	$12C_5^2$	$20C_3$	$15C_2$	i	$12S_{10}$	$12S_{10}^3$	$20S_6$	15σ
A_g	1	1	1	1	1	1	1	1	1	1
T_{1g}	3	$\frac{1}{2}(1+\sqrt{5})$	$\frac{1}{2}(1-\sqrt{5})$	0	−1	3	$\frac{1}{2}(1-\sqrt{5})$	$\frac{1}{2}(1+\sqrt{5})$	0	−1
T_{2g}	3	$\frac{1}{2}(1-\sqrt{5})$	$\frac{1}{2}(1+\sqrt{5})$	0	−1	3	$\frac{1}{2}(1+\sqrt{5})$	$\frac{1}{2}(1-\sqrt{5})$	0	−1
G_g	4	−1	−1	1	0	4	−1	−1	1	0
H_g	5	0	0	−1	1	5	0	0	−1	1
A_u	1	1	1	1	1	−1	−1	−1	−1	−1
T_{1u}	3	$\frac{1}{2}(1+\sqrt{5})$	$\frac{1}{2}(1-\sqrt{5})$	0	−1	−3	$-\frac{1}{2}(1-\sqrt{5})$	$-\frac{1}{2}(1+\sqrt{5})$	0	1
T_{2u}	3	$\frac{1}{2}(1-\sqrt{5})$	$\frac{1}{2}(1+\sqrt{5})$	0	−1	−3	$-\frac{1}{2}(1+\sqrt{5})$	$-\frac{1}{2}(1-\sqrt{5})$	0	1
G_u	4	−1	−1	1	0	−4	1	1	−1	0
H_u	5	0	0	−1	1	−5	0	0	1	−1

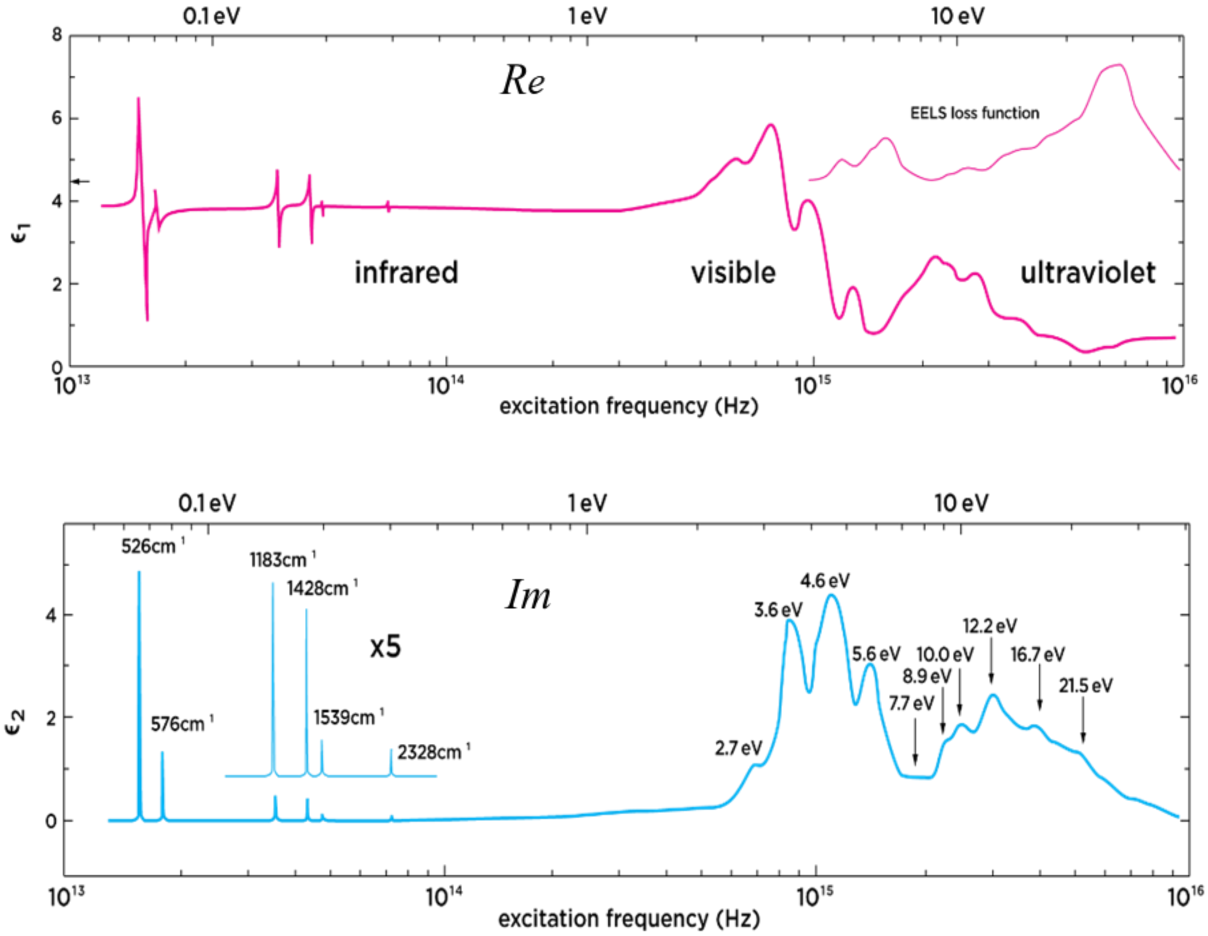

Figure S2: Real *Re*=$\varepsilon_1(\omega)$ and imaginary *Im*=$\varepsilon_2(\omega)$ frequency range of C_{60} molecule at room temperature. [Dresselhous, M.S.; Dresselhous, G.; Eklund, P.C. *Science of Fullerenes and Carbon Nanotubes*; Elsevier BV: Amsterdam, The Netherlands, 1996].

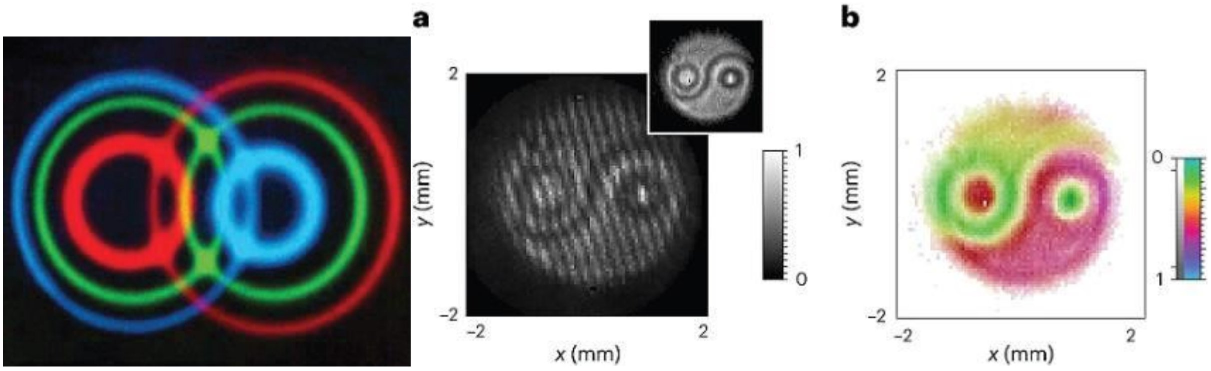

Figure S3: Yin-Yang in biphoton experiments: ***Left-***Visualization of quantum entanglement of two photons. ***Right a:*** Coincidence image of interference between a reference spontaneous parametric down-conversion state and a state obtained by a pump beam with the shape of a *Yīn* and *Yáng*. The inset scale is the same as in the main plot. ***Right b***: Reconstructed amplitude and phase structure of the image imprinted on the unknown pump. This quantum entanglement of two photons was experimentally visualized for the first time. Changes to the physical state of one particle in an entangled pair instantaneously cause the same change to occur in its partner – no matter how far apart they are ([Zia, D., Dhegihan, N., D'Errico, A., Sciarrino, F., Karimi, E., 2023). Interferometric imaging of amplitude and phase of spatial biphoton states, *Nature Photonics,* 1009–1016. https://doi.org/10.1038/s41566-023-01272-3].

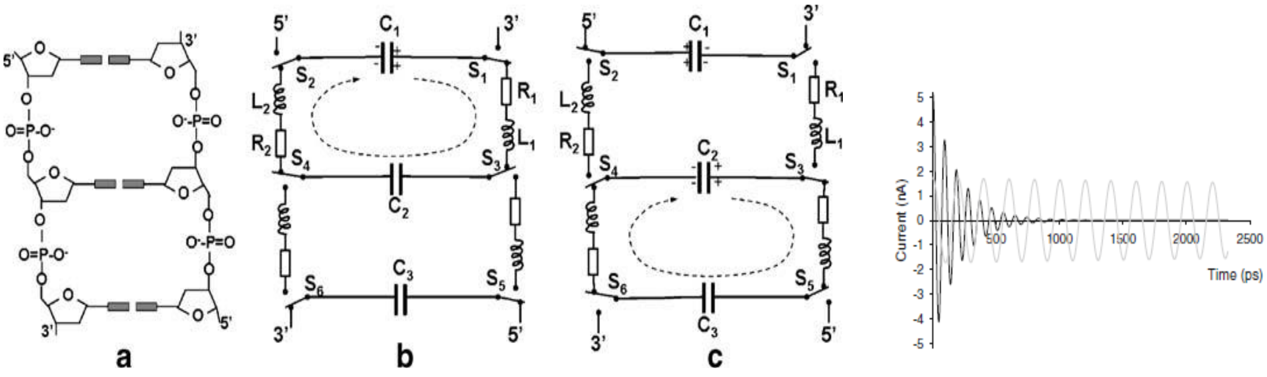

Figure S4: The hydrogen bonds oscillatory circuit of a DNA structure can be modeled as a circuit composed of multiple oscillatory *LC* circuits. In this model, non-covalent hydrogen bonds play the role of capacitance C, the phosphor group is *L* (inductance), and ribose is R (resistance). The *dashed arrows* indicate electric current directions, and the deoxyribose is represented by electric switches (a, b, c, left image). Model predictions for charge transfer of DNA circuit (A=T, C≡G) by fast stepwise under-damped oscillations in vitro (*black curve*) in contrast to slower stepwise simple harmonic oscillations in vivo (*gray curve*) where each cycle spans 0.34 nm in distance (right image). The circuit system transfers electric charges from one base pair to another in stepwise oscillatory processes, i.e., from capacitor C_1 to C_2 (left b) for about 200 *ps* (2×10^{-10} *s*). Because nucleotide bases have ionization potentials in the order of G<A<C<T and electron affinities in the order of C<T<G<A (disregarding the negative sign), it takes the least amount of energy to charge a G/C base pair in the polarity of *+G/C−* and requires the highest amount of energy to charge a *+T/A−* capacitor. This means that the capacitances of the base pairs are in the order of *+G/C−* > *+A/T−* > *+C/G−* > *+T/A−* polarities. [Kunming, Xu, Stepwise oscillatory circuits of DNA molecules. *J. Biol. Phys.* **2009**, *35*, 223–230. https://doi.org/10.1007/s10867-009-9149-9].
